# Small Intestinal Strangulation Secondary to Omental Adhesion Entrapment in a Dog

**DOI:** 10.1155/crve/3644983

**Published:** 2025-05-29

**Authors:** Alexandra Garrett, Hayley Gallaher, Maureen Spinner

**Affiliations:** Department of Small Animal Clinical Sciences, Michigan State University, East Lansing, Michigan, USA

## Abstract

A 6-year-old, male neutered, German Shorthaired Pointer was evaluated for acute onset of emesis, tenesmus, and restlessness. Abdominal radiographs suggested gastrointestinal obstruction or torsion. An exploratory laparotomy revealed an omental-to-mesenteric adhesion causing entrapment and strangulation of a segment of the jejunum, along with several other adhesions. The patient had undergone two previous laparotomies, which likely contributed to the extensive adhesion formation found intraoperatively. While postsurgical adhesion formation is a known complication in both human and veterinary medicine, this case is notable for the unusual origin of the adhesions arising from the omentum. Furthermore, the severity of strangulation resulting from this adhesion required more extensive surgery and posed a higher risk for long-term gastrointestinal complications, such as short bowel syndrome.

## 1. Introduction

Adhesion formation accounts for 75% of all cases of small bowel obstruction in human medicine [1]. Menzies in 1993 reported that the most common sites of adhesion formation resulting in intestinal obstruction in humans were small bowel to the previous site of surgery and small bowel to small bowel [2]. Only four human cases in which omental adhesions resulted in intestinal obstruction and/or strangulation have been reported. Of these four cases, three were associated with suspected congenital defects within the omentum, and a single case was reported to be due to a single omental adhesion secondary to a previous abdominal surgery [3–5].

In equine medicine, adhesions are the most frequent cause of postoperative colic and the second most common cause of repeated laparotomy [6, 7]. Within the equine veterinary literature, small intestinal strangulation from omental etiology specifically accounted for 2.3% of cases, of which 47% involved the omentum encircling a length of small intestine and 44% involved adhesions of the omentum to other structures [8].

Within the canine and feline literature, causes of intestinal entrapment and/or strangulation involving the omentum have only been previously reported with omental/mesenteric rents or rupture of the duodenocolic ligament, all sequelae to prior abdominal surgery [9–13]. However, no report of omental adhesion induced intestinal strangulation in these species has been reported to our knowledge.

This case report is aimed at describing the formation of an adhesion ring between a section of omentum and mesentery, which entrapped the jejunum and caused strangulation 3 years after a prophylactic gastropexy procedure. It details the clinical signs, diagnostic findings, surgical repair, and long-term outcome of this rare case of intestinal strangulation, believed to result from omental adhesion formation following prior prophylactic surgery.

## 2. Case Report

A 6-year-old, 28.7 kg (63.14 lbs), male neutered, German Shorthaired Pointer was presented to an emergency and referral hospital for a 2-day history of emesis, tenesmus, and restlessness. The patient was reported to have undergone two historic laparotomy procedures. The first was an exploratory laparotomy performed 5 years ago due to episodes of emesis and diarrhea, which revealed intestinal lymphadenopathy; however, no biopsies were obtained at that time. The second was a prophylactic gastropexy performed 3 years prior following an episode of food bloat. Despite surgical interventions, the dog was reported to have monthly gastrointestinal upset in the form of emesis and inappetence, which was routinely treated with prednisone (0.2 mg/kg orally) once daily until resolution of clinical signs.

The dog was presented to the referring veterinarian 1 day prior, and abdominal radiographs were performed, which demonstrated areas of gas distention within the small intestines. The dog was discharged with medical management therapy, including methocarbamol (18 mg/kg orally every 8 h) and prednisone (0.7 mg/kg orally every 12 h).

**Figure 1:**
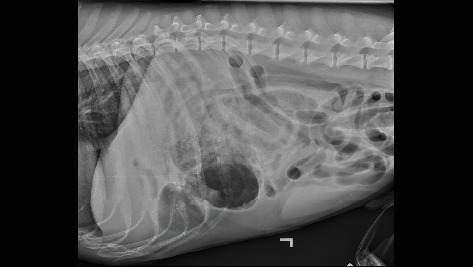
Left lateral radiograph highlighting heterogenous material in pyloric antrum.

**Figure 2:**
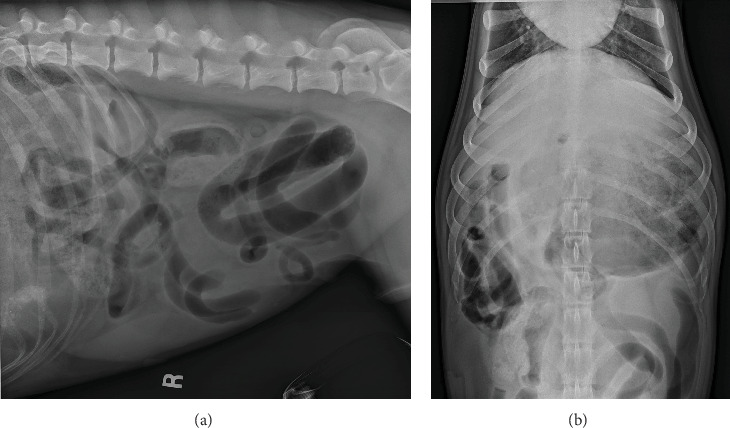
Right lateral radiograph highlighting distention and (a) stacking of small intestines in the caudal abdomen and (b) stacking of intestines in right cranial abdomen.

On referral presentation, the patient was hypertensive (180 mmHg with a Doppler blood pressure measuring device) with a mildly distended and severely painful abdomen upon palpation. A venous blood gas, complete blood count, and serum biochemistry were obtained and revealed hyperlactatemia (3.8, Ref. 0.6–3.3 mmol/L), hypochloremia (102, Ref. 110–117 mmol/L), hypocalcemia (9, Ref. 9.5–10.8 mmol/L), elevated total bilirubin (0.4, Ref. 0.1–0.3 mg/dL), elevated alkaline phosphatase (ALP) (164, Ref. 10–92 U/L), elevated alanine aminotransferase (ALT) (51, Ref. 16–41 U/L), leukocytosis (27.2, Ref. 4.6–10.7 × 10e^3^ U/L), neutrophilia with a left shift (segmented neutrophils 23.7, Ref. 2.7–7.8 × 10e^3^ U/L, band neutrophils (2.2, Ref. 0.0–0.1 × 10e^3^ U/L), lymphopenia (0.3, Ref. 0.6–5.0 × 10e^3^ U/L), and monocytosis (1.1, Ref. 0.1–0.8 × 10e^3^ U/L). Packed cell volume and total solids were elevated at 60% and 7.8 g/dL, respectively. The patient was administered methadone (0.2 mg/kg IV) and midazolam (0.2 mg/kg IV) for orthogonal abdominal radiographs to be performed. Gastric distension with heterogenous soft tissue material within the pyloric antrum (Figure 1) and evidence of small intestinal mechanical obstruction including small intestinal distention and stacking were identified (Figure 2). Radiographic differentials included foreign body obstruction, stricture with an intussusception or underlying neoplasia, adhesion formation, and less likely intestinal thromboemboli. A diagnosis of mesenteric torsion was considered less likely due to the incomplete distention of the intestinal tract. Following radiographs, the dog was treated with a 22 mL/kg IV lactated Ringer's solution (LRS) bolus over 15 min, and a repeat blood pressure had decreased to 165 mmHg, likely secondary to sedation in hospital.

**Figure 3:**
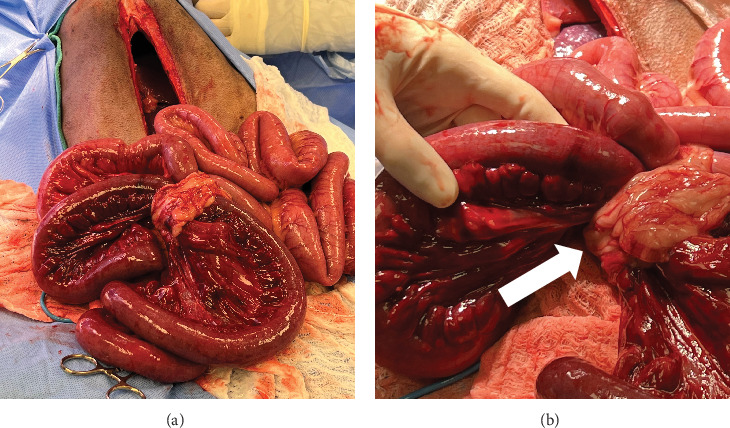
Intraoperative pictures of (a) compromised jejunum and ileum and (b) omental adhesion forming a ring with secondary strangulation of intestines.

Exploratory laparotomy was recommended and pursued, and a moderate amount of serosanguineous transudate was present upon entry into the abdomen and actively suctioned. Multiple omental adhesions were present crossing the liver and stomach, covering and obstructing the historic prophylactic gastropexy site, and foreign material was palpated within the pyloric antrum. These adhesions were suspected to have formed as a result of the historically performed prophylactic gastropexy. Additionally, an omental to mesenteric ring adhesion resulting in entrapment and strangulation of a segment of the jejunum was identified (Figure 3). The omental adhesion was transected with a LigaSure Atlas (Covidien, Mansfield, Massachusetts, United States), and a functional end-to-end resection and anastomosis was performed at the transition of healthy and necrotic bowel using an Endo GIA 60 mm stapler (EGIA60AVM, Covidien, Mansfield, Massachusetts, United States), which resulted in the removal of 3 ft of small bowel. The anastomosis was reinforced with simple interrupted crotch sutures and oversewing of the transverse line in a simple continuous suture pattern using 3-0 polydioxanone (PDSII, Ethicon, Guaynabo, Puerto Rico). Sequentially, a partial omentectomy to enable visualization of the historic gastropexy site was performed. This revealed that the historic gastropexy had been performed between the body wall and gastric body, resulting in an acute curvature of the pylorus. A partial gastrectomy to remove the scar was performed and utilized for foreign body retrieval which consisted of a large amount of grass. The gastrostomy was apposed in two layers with a simple continuous pattern in the mucosa–submucosa layer and Cushing's pattern in the seromuscular layer with 3–0 polydioxanone (PDSII, Ethicon LLC, Guaynabo, Puerto Rico). Finally, a prophylactic right sided gastropexy between the pyloric antrum and body wall was performed using 2-0 polydioxanone (PDSII, Ethicon LLC, Guaynabo, Puerto Rico). Warm sterile saline was used to lavage the abdomen multiple times and was removed via Poole suction. The linea alba was closed with 0 polydioxanone (PDSII, Ethicon LLC, Guaynabo, Puerto Rico) in a simple continuous pattern. The subcutaneous layer was closed with 2-0 Poliglecaprone 25 (Monocryl, Ethicon LLC, Guaynabo, Puerto Rico) in a simple continuous pattern with intermittent fascial tacking. The subcutaneous tissue was injected with bupivacaine liposome injectable suspension (Nocita, Elanco US Inc., Greenfield, Indiana, United States) throughout the length of the incision. The skin was apposed in a continuous intradermal pattern with 3–0 Poliglecaprone 25 (Monocryl, Ethicon LLC, Guaynabo, Puerto Rico).

The patient recovered without complications and was monitored in the hospital for 3 days before being discharged for at-home care. Three weeks later, the dog returned for a scheduled postoperative check-up. The laparotomy incision had healed well, though chronic loose stools were noted. As of the 3-month follow-up, the dog was doing well overall, with only persistent soft stools and no other concerns.

## 3. Discussion

Abdominal surgery is commonly performed in small animal medicine for a variety of reasons, ranging from elective procedures to life-saving interventions. While each abdominal surgery carries its own set of risks, adhesion formation is an inherent postoperative complication for all, and efforts should be made to minimize adhesion development during surgery. Adhesion formation occurs secondary to damage to or inflammation of the peritoneal mesothelium. This triggers an immediate procoagulant response, characterized by the release of serofibrinous exudate and the accumulation of fibrin. Within hours of injury, fibrin forms a matrix that facilitates cellular migration for tissue repair, which can lead to adhesions between nearby structures [14]. Eventually, fibrinolysis is activated through tissue plasminogen activator (tPA). However, when the mesothelium is injured or inflamed, such as in the case of surgical manipulation, ischemia at the site reduces tPA levels, impairing fibrinolysis and promoting the accumulation of fibrous tissue. This process contributes to the formation of adhesions in surrounding viscera [15]. Multiple techniques have been shown to be effective in reducing adhesion formation, including meticulous surgical technique, the use of antibiotics and nonsteroidal anti-inflammatory drugs, intraoperative peritoneal lavage, liquid barriers such as heparin, strategies to prevent abdominal contamination, and autologous peritoneal grafting [16–20]. As the frequency of postoperative adhesion complications increases in small animals, greater emphasis should be placed on the routine and prophylactic use of these strategies to minimize adhesion formation.

As noted earlier, adhesion formation is well-documented in human medicine. However, the specific incidence of postoperative adhesion formation in small animals is unknown and likely underreported due to shorter lifespans, limited routine diagnostic workups, and infrequent necropsy. This case highlights an example of a severe consequence of adhesion formation, secondary to surgical manipulation, and emphasizes the importance of mitigating their formation, an aspect that may not always be emphasized in general practice. In fact, studies in equine surgery show that around 15% of horses undergoing abdominal surgery develop adhesions. However, many of the prevention protocols deemed effective in the literature are not routinely applied in clinical practice [21]. While this has not been surveyed in small animal surgery, a similar neglect of adhesion prevention is likely, given that the incidence of adhesion-related complications is reported to be lower than in equine medicine. Therefore, adhesion complication risk should be thoroughly discussed and mitigated, given the frequency of laparotomies practiced in small animal general practice ranging from cystotomies, gastrotomies, gastropexies, and ovariohysterectomies.

In addition, the chronic, fluctuating gastrointestinal symptoms and eventual acute abdominal presentation in this case and in another case report highlight the insidious nature of adhesions and the broad range of complications they can cause [20]. A more thorough diagnostic work-up of gastrointestinal upset in patients with a history of abdominal surgery could be beneficial to consider iatrogenic causes, such as adhesion formation, rent formation, and improper placement of a gastropexy site. Doing so might have led to earlier intervention and a less extensive surgical resection for this patient.

It is important to note that this case is not intended to discourage abdominal surgery. Instead, it underscores the importance of informing owners about the risks of abdominal surgery and the potential complications that may arise along with the emphasis on practicing adhesion mitigation techniques intraoperatively. Additionally, this case serves as a reminder for clinicians to consider adhesion formation as a differential diagnosis in patients with new gastrointestinal signs and a history of abdominal surgery, warranting further investigation. While this is the first reported case of small intestinal strangulation due to omental entrapment, it emphasizes the importance of gentle tissue handling during surgery, the use of techniques to mitigate their formation, and raises awareness about potential variations in adhesion formation across different species.

## Data Availability

All data are available within the manuscript.
